# Machine learning prediction of postoperative unplanned 30-day hospital readmission in older adult

**DOI:** 10.3389/fmolb.2022.910688

**Published:** 2022-08-10

**Authors:** Linji Li, Linna Wang, Li Lu, Tao Zhu

**Affiliations:** ^1^ Department of Anesthesiology, West China Hospital, Sichuan University and The Research Units of West China (2018RU012), Chinese Academy of Medical Sciences, Chengdu, China; ^2^ Department of Anesthesiology, The Second Clinical Medical College, North Sichuan Medical College, Nanchong Central Hospital, Nanchong, China; ^3^ College of Computer Science, Sichuan University, Chengdu, China

**Keywords:** machine learning, unplanned hospital readmission, surgery, prediction, elderly

## Abstract

**Background:** Although unplanned hospital readmission is an important indicator for monitoring the perioperative quality of hospital care, few published studies of hospital readmission have focused on surgical patient populations, especially in the elderly. We aimed to investigate if machine learning approaches can be used to predict postoperative unplanned 30-day hospital readmission in old surgical patients.

**Methods:** We extracted demographic, comorbidity, laboratory, surgical, and medication data of elderly patients older than 65 who underwent surgeries under general anesthesia in West China Hospital, Sichuan University from July 2019 to February 2021. Different machine learning approaches were performed to evaluate whether unplanned 30-day hospital readmission can be predicted. Model performance was assessed using the following metrics: AUC, accuracy, precision, recall, and F1 score. Calibration of predictions was performed using Brier Score. A feature ablation analysis was performed, and the change in AUC with the removal of each feature was then assessed to determine feature importance.

**Results:** A total of 10,535 unique surgeries and 10,358 unique surgical elderly patients were included. The overall 30-day unplanned readmission rate was 3.36%. The AUCs of the six machine learning algorithms predicting postoperative 30-day unplanned readmission ranged from 0.6865 to 0.8654. The RF + XGBoost algorithm overall performed the best with an AUC of 0.8654 (95% CI, 0.8484–0.8824), accuracy of 0.9868 (95% CI, 0.9834–0.9902), precision of 0.3960 (95% CI, 0.3854–0.4066), recall of 0.3184 (95% CI, 0.259–0.3778), and F1 score of 0.4909 (95% CI, 0.3907–0.5911). The Brier scores of the six machine learning algorithms predicting postoperative 30-day unplanned readmission ranged from 0.3721 to 0.0464, with RF + XGBoost showing the best calibration capability. The most five important features of RF + XGBoost were operation duration, white blood cell count, BMI, total bilirubin concentration, and blood glucose concentration.

**Conclusion:** Machine learning algorithms can accurately predict postoperative unplanned 30-day readmission in elderly surgical patients.

**Trial registration:** http://www.chictr.org.cn/showproj.aspx?proj=35795, ChiCTR, ChiCTR1900021290

## Background

The unplanned hospital readmission rate is one of the most widely used indicators to assess hospital care quality (Gupta and Fonarow, 2018). Due to its substantial contribution to medical resource costs, unplanned hospital readmission is increasingly recognized as an important public health concern, especially in developed countries (Jencks et al., 2009; Axon and Williams, 2011). Geriatric surgical patients, vulnerable to chronic illnesses, are at higher risk of unplanned hospital readmission with compounded factors. Although not all of these readmissions are preventable, it is critical to propose an effective framework for their early identification. A substantial body of models exists to identify patients at risk for unplanned readmission (Miotto et al., 2016; Kansagara et al., 2011; van Walraven et al., 2012; ohnson et al., 2019). However, most of them were created based on a specific disease cluster and cannot be extrapolated to the entire postoperative population, particularly elderly surgical patients (Ali and Gibbons, 2017; Ko et al., 2020; Kong and Wilkinson, 2020; Mišić et al., 2020; Sander et al., 2020; Shebeshi et al., 2020; Wasfy et al., 2020; Amritphale et al., 2021).

Recently, machine learning (ML) algorithms were considered to be potential tools for developing clinical predictive models because of their ability to deal with multidimensional datasets and make accurate predictions (Deo, 2015; Jordan and Mitchell, 2015). Since ML algorithms can process nonlinear relationships and interactions between predictors, they may be increasingly used in medical modeling. In this study, we aimed to investigate if ML-based algorithms can accurately predict postoperative unplanned 30-day readmission in an elderly surgical patient cohort using input features, such as demographic, comorbidity, laboratory, surgical, and medication data.

## Methods

### Data extraction

**FIGURE 1:**
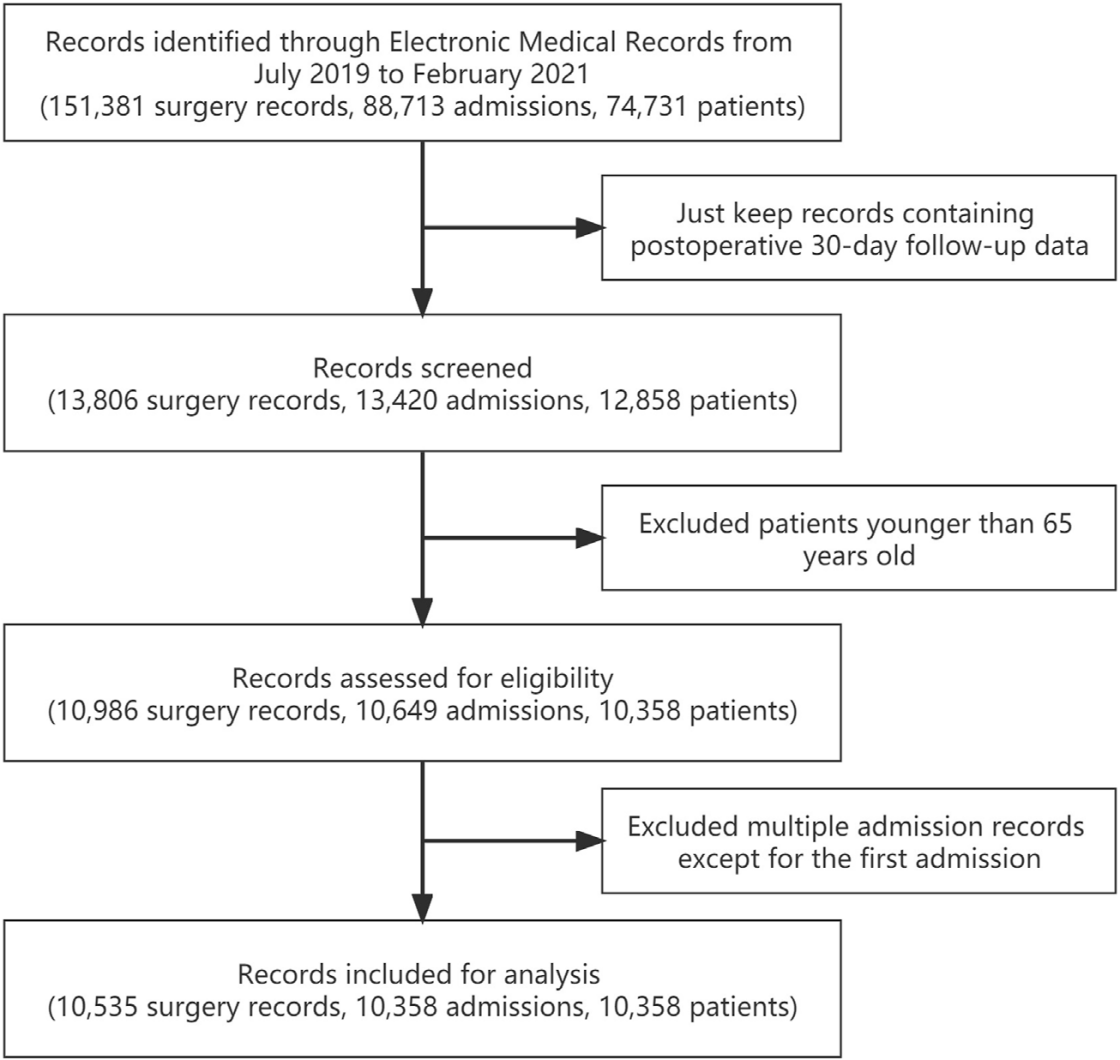
Flow chart of inclusion and exclusion process for overall data set.

This study has been registered in the Chinese Clinical Trial Registry (ChiCTR-1900021290), and ethical approval was obtained from the Ethical Review Board of West China Hospital, Sichuan University, China. All the relevant clinical data were prospectively collected during the course of our routine anesthesia risk assessment, intraoperative records, and postoperative follow-up using a structured data schema designed by our institution. We extracted perioperative information of elderly patients older than 65 who underwent surgeries under general anesthesia in West China Hospital, Sichuan University from July 2019 to February 2021. For patients who had multiple admission records, we only included their first admissions for analysis. Meanwhile, for patients who underwent multiple surgeries during a single hospitalization, we included all their surgeries for analysis. A flow chart describing the inclusion and exclusion process is shown in Figure 1.

### Model endpoint definition

The label “postoperative 30-day unplanned readmission” was defined as follows: readmission due to the same surgical disease or postoperative complications within 30 days postoperatively in an unplanned fashion. Our professional follow-up personnel collected this information by telephone 30 days after surgery.

### Data preprocessing

**FIGURE 2:**
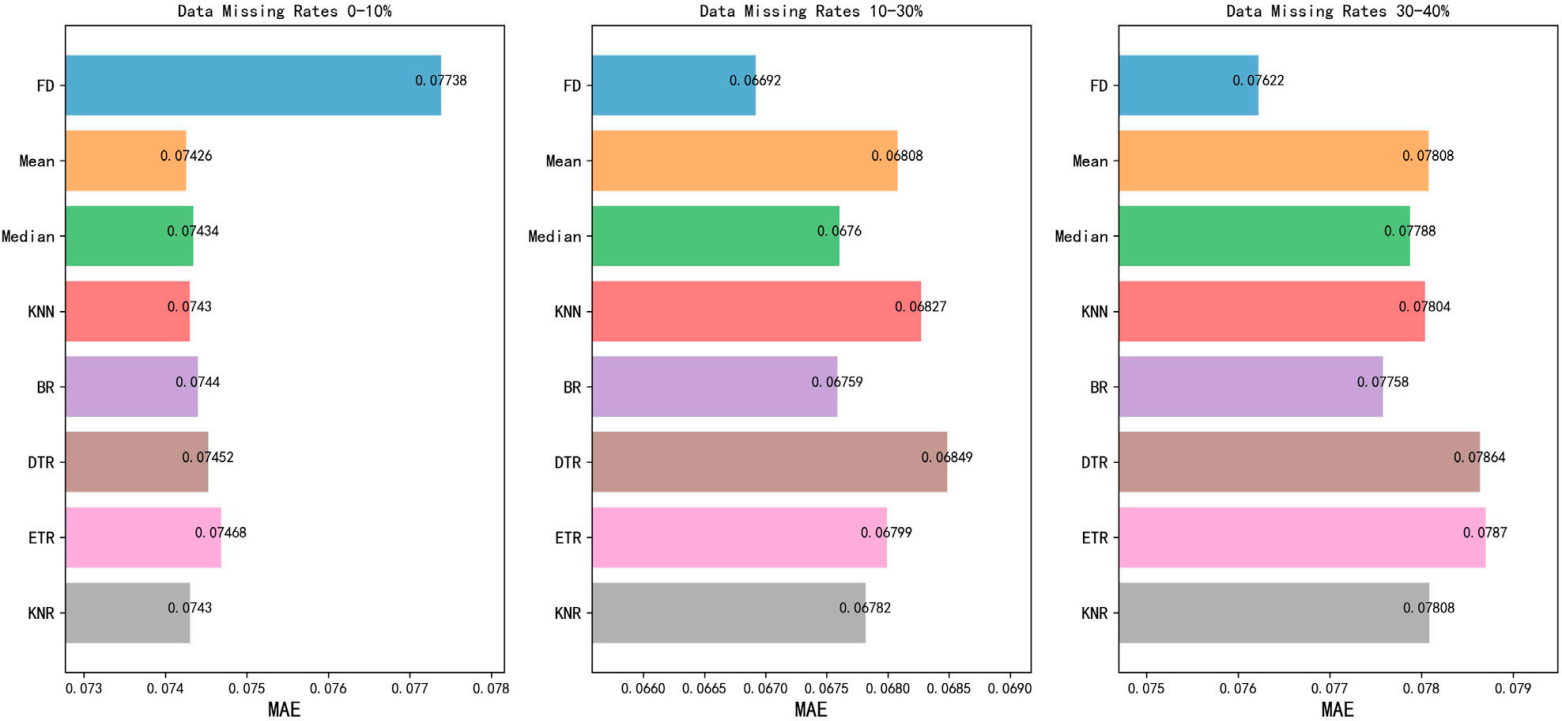
Imputation techniques in different missing data groups. FD, Full data; KNN, k nearest neighbor; BR, BayesianRidge; DTR, DecisionTreeRegressor; ETR, ExtraTreesRegressor; KNR, KNeighborsRegressor; MAE, Mean Absolute Error. BayesianRidge performed the best with the lowest MAE among all imputation techniques.

There were few admissions with missing data. Variables with a missing data rate greater than 30% were not included for model development. For numeric variables with a missing data rate less than 5%, the median of each variable was used for imputation. For numeric variables with a missing data rate between 5% and 30%, we performed various imputation techniques using mean absolute error (MAE) scores as estimated metrics for comparison. To estimate the score on an original full dataset, we excluded all missing value rows and randomly removed some values to create a new version of the dataset with artificially missing data. Then, we compared the performance of the random forest (RF) regressor on the complete original dataset with that on the altered dataset that used different imputation techniques. The comparison results presented in Figure 2 showed that we could find the lowest MAE to impute the missing values.

Considering the extreme imbalanced classification between the readmitted samples and non-readmitted samples (the readmission rate is only 3.36%), we both oversampled and undersampled the training set using the Synthetic Minority Over-sampling Technique (SMOTE) and Edited Nearest Neighbors (ENN). The SMOTE generated noisy samples by interpolating new points between marginal outliers and inliers, while ENN cleaned the space resulting from oversampling. Utilizing the SMOTE + ENN (SMOTEENN) algorithm provided by the imbalanced-learn *Python* library, we achieved a more balanced data distribution of readmitted samples and non-readmitted samples (Lemaître et al., 2017).

Our data were randomly divided into a training set and a test set according to a 70–30 split. We estimated models based on the training data (70%) and evaluated models based on the test data (30%). Each split was carried out to preserve the proportion of readmitted and not readmitted cases in the entire dataset. This random split was repeated ten times.

### Feature selection

We focused on features that are easily accessible and not only available after discharge. For the preoperative laboratory data, we kept the last value prior to surgery. Before feature selection, we obtained 145 initial available variables. In model development, variable selection reduces the number of attributes and allows the selection of a subset of relevant features. Generally, there are three classes of optimal feature selection algorithms as follows: filter, wrapper, and embedded methods. In this study, we used the wrapper method because it can measure the usefulness of features based on the classifier performance through the search process, where different combinations of features are evaluated and compared by scores based on predictive model accuracy (Chandrashekar and Sahin, 2014).

To eliminate irrelevant, weakly relevant, or redundant features and reduce model overfitting as well as improve model generalization ability, we used a multilayer perceptron (MLP) as an estimator to implement a genetic algorithm (GA), which is a stochastic search algorithm based on the mechanics of evolution and natural selection (Torkamanian-Afshar et al., 2021). GA uses three operators, that is, selection, crossover, and mutation to improve the quality of solutions. We used Distributed Evolutionary Algorithms in *Python* to implement GA, while the function returns the optimal setting of feature selection as a binary array with the best accuracy score (Rainville et al., 2014). The independent probability for each attribute to be flipped was 0.1 in multiple flip-bit mutations. Tournament selection was set as the selection operator with a tournament size of 3. The population size was 100, the crossover probability was 0.5, and the mutation probability was 0.2.

The full list of features includes demographic data (e.g., age, gender, and body mass index [BMI]), available obtained laboratory tests prior to surgery (e.g., glucose concentration and oxygen saturation), descriptive intraoperative vital signs (e.g., systolic blood pressure), comorbidity (e.g., hypertension), and surgery descriptions (e.g., surgery type and anesthesia).

### Model creation, training, and testing

This study considered different widespread types of models, that is, logistic regression, MLP, RF, extreme gradient boosting (XGBoost), and light gradient boosting machine (LGBM). The latter three are bagging or boosting ensemble learning algorithms. XGBoost is an optimized distributed gradient boosting library designed to have strong predictive power. It does not build the full tree structure but builds it greedily. It provides a parallel tree boosting that solves scientific problems, such as regression, classification, and ranking, in a fast and accurate way. LGBM is a high-performance gradient lifting framework that is based on a decision tree. Thus, it splits the tree leaf-wise with the simplest fit, whereas other boosting algorithms split the tree depth- or level-wise instead of leaf-wise. LGBM is quick because it uses a histogram-based algorithm that quickens the training procedure. We calculated MAEs as weights to combine RF and XGBoost into a hybrid model.

One of the advantages of using the abovementioned algorithms is that we can easily calculate the scores for all the input features, which represent the importance of each feature. A specific feature with a higher score means that it will have a larger effect on the model prediction. Random Forest Classifier, Logistic Regression, and MLP Classifier used in this study are from Scikit-learn. The XGB Classifier and LGBM Classifier were implemented using the xgboost and lightgbm packages (*Python* Software Foundation, 9450 SW Gemini Dr., ECM# 90772, Beaverton, OR 97008, United States) separately.

Model hyperparameters were set before training to improve the performance of the algorithms. We used RandomizedSearchCV and GridSearchCV provided by Scikit-learn. Five-fold cross-validation was applied to the training set, meaning that we calculated the average metrics while each of the five partitions was treated only once as a test set and four times as a training set. Before parameter optimization, all model classifier parameters were set to default values. We first used a random search with 200 iterations, and then a smaller range was determined based on the parameter selected in the previous step, and Grid Search worked with a small number of hyperparameters.

We used block bootstrapping to generate confidence intervals (CIs) for the performance metrics on the test set. Rather than randomly sampling procedures, we randomly sampled patients 1,000 times, included all predictions in the bootstrap sample, and sorted the performance metrics of each bootstrap sample.

### Evaluation metrics

Model performance was assessed using the following metrics: area under the ROC curve (AUC), accuracy, precision, recall, and F1 score. ROC curve, as a visualization tool, can infer model performance by illustrating the relationship between precision and recall as we vary the threshold for selecting positives. Each time a different threshold was selected, a set of false-positive and true-positive rates were obtained. The calibration of the model was evaluated by Brier score and calibration plots. The 95% CIs of the abovementioned indicators were calculated through 1,000 repeated sampling. A feature ablation analysis was performed, and the change in AUC with the removal of each feature was then assessed to determine feature importance.

## Results

### Characteristics of the patients

Inclusion and exclusion criteria were strictly followed during the entire screening process. A flow chart indicating the inclusion and exclusion process is shown in Figure 1. Finally, a total of 10,358 elderly patients were included. The overall 30-day unplanned readmission rate was 3.36%. The demographic data and surgery-related information of patients are shown in Table 1.

**TABLE 1:** Summary of demographic characteristics and perioperative data in this cohort.

Variables	Training set	Testing set
Patients, n	**6,916**	**3,442**
Surgery, n (%)	**7,058(67.0)**	**3,477(33.0)**
Age (SD)	**72.1(5.8)**	**71.9(5.7)**
Female, n (%)	**2,990(43.2)**	**1,507(43.8)**
Readmission, n (%)	**237(3.36)**	**117(3.36)**
ASA		
**Ⅰ, n (%)**	**11(0.16)**	**5(0.14)**
Ⅱ, **n (%)**	**3,426(48.54)**	**1,667(47.94)**
Ⅲ, **n (%)**	**3,531(50.03)**	**1764(50.73)**
Ⅳ, **n (%)**	**84(1.19)**	**39(1.12)**
**Ⅴ, n (%)**	**6(1.0)**	**2(0.06)**
Surgery type		
Abdominal, n (%)	**3,711(52.58)**	**1782(51.25)**
Orthopedic, n (%)	**1,246(17.65)**	**673(19.36)**
Thoracic, n (%)	**636(9.01)**	**304(8.74)**
Cardiac, n (%)	**295(4.18)**	**163(4.69)**
Neuro, n (%)	**11(0.16)**	**5(0.14)**
Other, n (%)	**1,159(16.42)**	**550(15.82)**

The values in bold mean that they have the best performance in the metrics compared with all the other ML algorithms.

### Model performance

**FIGURE 3:**
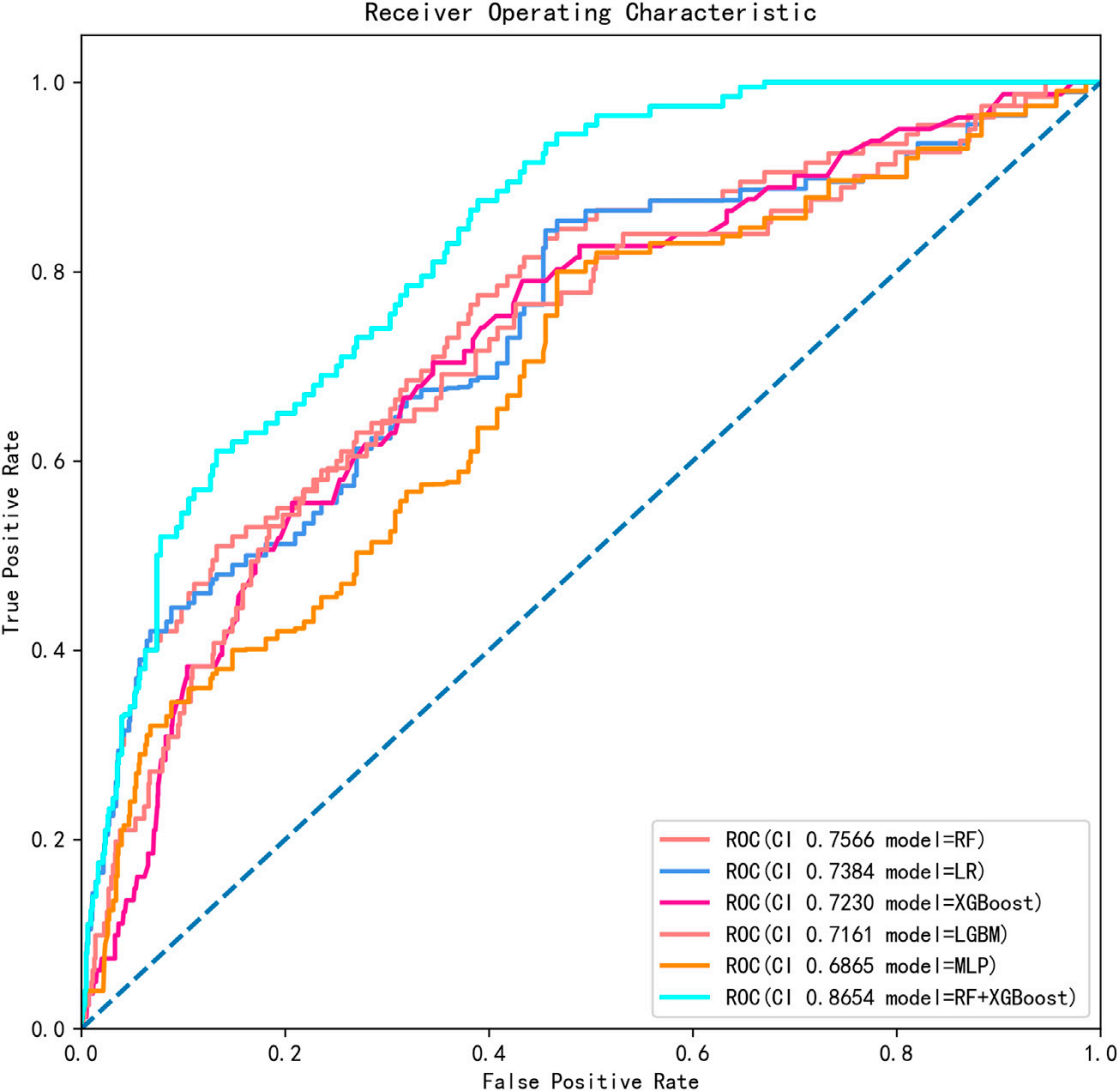
The ROC curves and AUCs of six ML algorithms predicting postoperative unplanned 30-day hospital readmission in this cohort. ROC, receiver operating characteristic; AUC, area under the curve; RF, random forest; LR, logistic regression; XGBoost, eXtreme Gradient Boosting; LGBM, Light Gradient Boosting Machine; MLP, Multilayer Perceptron.

**FIGURE 4:**
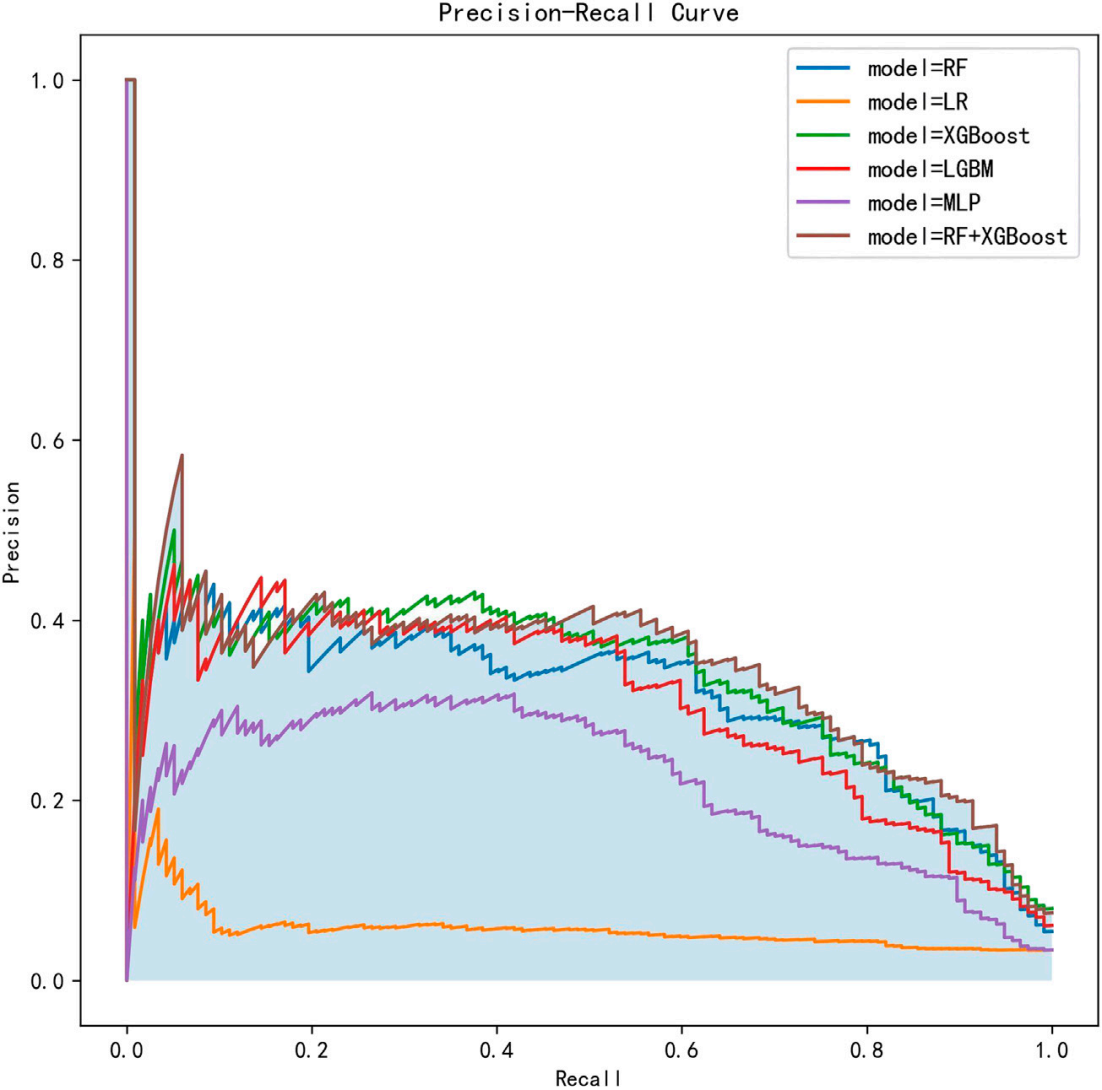
The P-R curves of six ML algorithms predicting postoperative unplanned 30-day hospital readmission in this cohort. P-R, Precision-Recall; RF, random forest; LR, logistic regression; XGBoost, eXtreme Gradient Boosting; LGBM, Light Gradient Boosting Machine; MLP, Multilayer Perceptron.

The AUCs of the six ML algorithms predicting postoperative 30-day unplanned readmission ranged from 0.6371 to 0.7686 including all features (Table 2) and from 0.6865 to 0.8654 including selected features (Table 3). The RF + XGboost classifier including selected features overall performed the best with an AUC of 0.8654 (95% CI, 0.8484-0.8824), the accuracy of 0.9868 (95% CI, 0.9834–0.9902), the precision of 0.3960 (95% CI, 0.3854–0.4066), recall of 0.3184 (95% CI, 0.259–0.3778), and F1 score of 0.4909 (95% CI, 0.3907–0.5911) (Table 3); The ROC curves of all the six ML algorithms predicting postoperative unplanned 30-day hospital readmission are shown in Figure 3, and the Precision-Recall (P-R) curves of all the six ML algorithms are also shown in Figure 4.

**TABLE 2:** Performance of classification models including all features.

Model	AUC (95% CI)	Accuracy (95% CI)	Precision (95% CI)	Recall (95% CI)	F1 (95% CI)
RandomForest	0.7105 (0.6860–0.7350)	**0.9620(0.9610–0.9630)**	**0.3501(0.3000–0.4001)**	0.0120 (0.0110–0.0130)	0.0240 (0.0230–0.0250)
LogisticRegression	0.7145 (0.7110–0.7180)	0.9580 (0.9570–0.9590)	0.2160 (0.1820–0.2500)	0.0250 (0.0240–0.0260)	0.0442 (0.0431–0.0452)
XGBoost	0.6795 (0.6750–0.6840)	0.9606 (0.9601–0.9611)	0.2665 (0.2000–0.3333)	0.0125 (0.0120–0.0130)	0.0237 (0.0233–0.0240)
LGBM	0.6725 (0.6690–0.6760)	0.9595 (0.9590–0.9600)	0.2085 (0.1670–0.2500)	0.0125 (0.0120–0.0130)	0.0230 (0.0220–0.0240)
MLP	0.6371 (0.5741–0.7000)	0.9475 (0.9380–0.9570)	0.1621 (0.0630–0.2611)	0.0740 (0.0250–0.1230)	0.0920 (0.0350–0.1490)
Random + XGBoost	**0.7686(0.7396–0.7977)**	0.9524 (0.9523–0.9525)	0.3471 (0.3315–0.3627)	**0.1030(0.0950–0.1110)**	**0.1120(0.1100–0.1140)**

The values in bold mean that they have the best performance in the metrics compared with all the other ML algorithms.

**TABLE 3:** Performance of classification models including selected features.

Model	AUC (95% CI)	Accuracy (95% CI)	Precision (95% CI)	Recall (95% CI)	F1 (95% CI)
RandomForest	0.7566 (0.7481–0.7651)	0.9862 (0.9838–0.9885)	0.3950 (0.3900–0.4000)	0.3089 (0.1600–0.4578)	0.4287 (0.3952–0.4622)
LogisticRegression	0.7384 (0.7357–0.7411)	0.9503 (0.9474–0.9532)	0.2936 (0.2252–0.3620)	0.155 (0.1223–0.1878)	0.1957 (0.1406–0.2508)
XGBoost	0.7230 (0.7136–0.7324)	0.9862 (0.9835–0.9889)	**0.3977(0.3854–0.4100)**	**0.3289(0.2622–0.3955)**	0.4371 (0.3931–0.4812)
LGBM	0.7161 (0.6778–0.7544)	0.9867 (0.9855–0.988)	0.3882 (0.3763–0.4000)	0.3261 (0.2945–0.3578)	0.4385 (0.4197–0.4573)
MLP	0.6865 (0.6504–0.6226)	0.9744 (0.9711–0.9778)	0.2683 (0.2226–0.3140)	0.2434 (0.1568–0.3300)	0.2653 (0.6026–0.3281)
Random + XGBoost	**0.8654(0.8484–0.8824)**	**0.9868(0.9834–0.9902)**	0.3960 (0.3854–0.4066)	0.3184 (0.259–0.3778)	**0.4909(0.3907–0.5911)**

The values in bold mean that they have the best performance in the metrics compared with all the other ML algorithms.

The Brier score of the RF + XGboost model predicting postoperative 30-day unplanned readmission was 0.0372 (95% CI, 0.0371–0.0372), showing the best calibration capability among all the ML algorithms (Table 4).

**TABLE 4:** Calibration of classification models including selected features.

Model	Brier Score (95% CI)
RandomForest	0.0383 (0.0377–0.0388)
LogisticRegression	0.0399 (0.0394–0.0403)
XGBoost	0.0389 (0.0386–0.0392)
LGBM	0.0377 (0.0375–0.0379)
MLP	0.0464 (0.0408–0.0519)
Random + XGBoost	**0.0372(0.0371–0.0372)**

The values in bold mean that they have the best performance in the metrics compared with all the other ML algorithms.

### Feature importance

**FIGURE 5:**
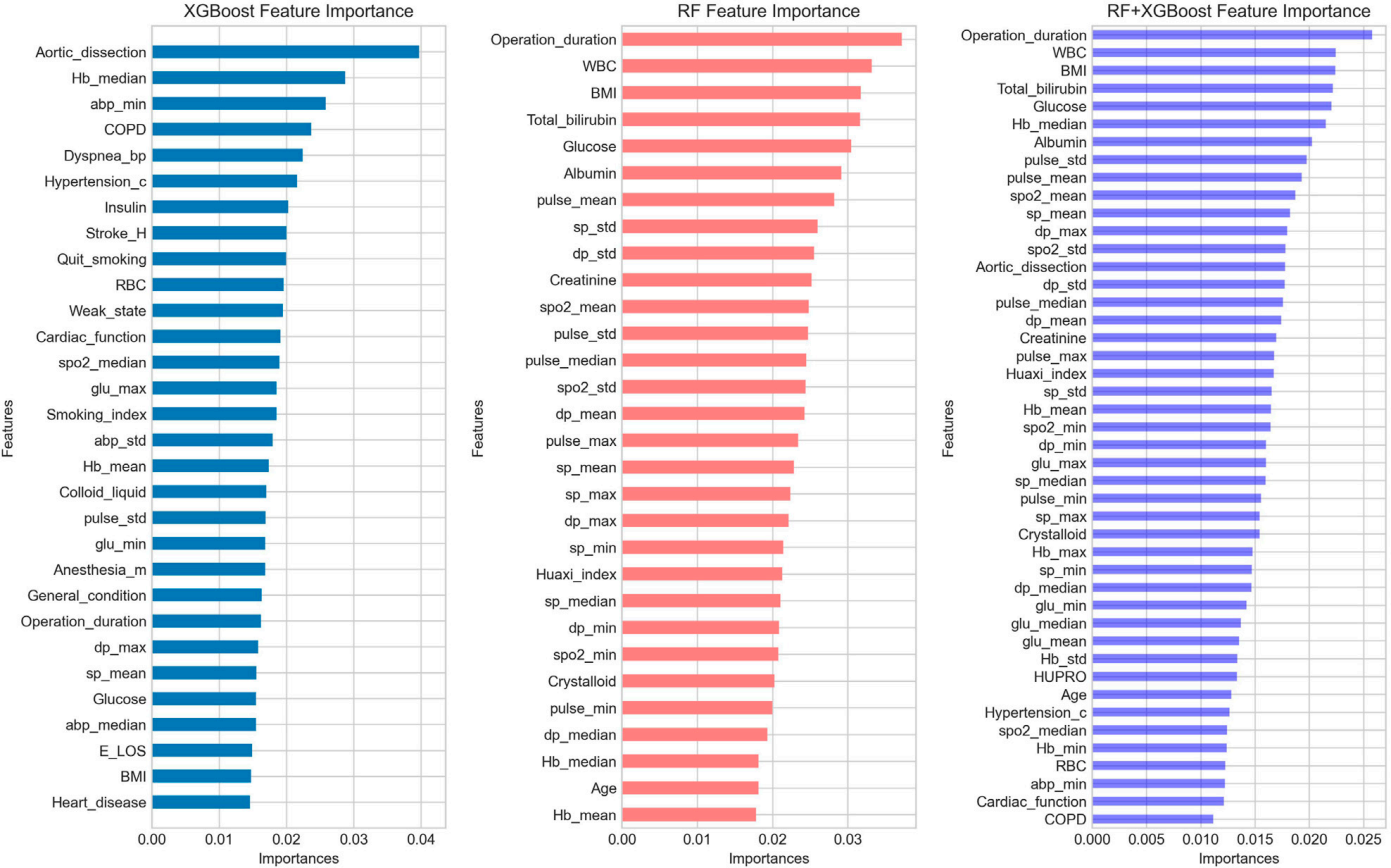
The feature importance of three ML algorithms predicting postoperative unplanned 30-day hospital readmission. RF, random forest; XGBoost, eXtreme Gradient Boosting.

After performing a feature ablation analysis, we found that the five most important features of the RF + XGboost model were operation duration, white blood cell count, BMI, total bilirubin concentration, and blood glucose concentration. Figure 5 presents the feature importance of three models (RF、XGboost, and RF + XGboost) predicting postoperative unplanned 30-day hospital readmission.

## Discussion

We used five ML models separately and one hybrid model to predict the 30-day postoperative unplanned readmission of elderly patients. To analyze the performance of the proposed framework, we investigated the advantages and benefits of the proposed model over traditional ML models. Among all the algorithms, the RF + XGboost hybrid model generally performed relatively better, with an AUC of 0.8654 (95% CI, 0.8484–0.8824) and a Brier score of 0.0372 (95% CI, 0.0371–0.0372). For a single ML algorithm, RF nearly had the best performance in predicting the 30-day postoperative unplanned readmission, which has previously been reported (Peng et al., 2010; Hsieh et al., 2011; Alickovic and Subasi, 2016; Gowd et al., 2019). In addition, all ML models tended to perform similarly or better than the traditional approach (van Walraven et al., 2010; Cotter et al., 2012; Donzé et al., 2013; Low et al., 2017).

In the RF + XGboost model, the five most important features were operation duration, white blood cell count, BMI, total bilirubin concentration, and glucose concentration. Long duration of surgery is an important factor resulting in multiple postoperative complications, including unplanned 30-day postoperative readmission (Phan et al., 2017; Polites et al., 2017). Increased white blood cell count usually indicates an increased likelihood of infection. Postoperative infection is also an important reason for unplanned readmissions, such as lung infection requiring anti-infective treatment or wound infection requiring readmission for debridement or surgery. An increase in BMI is closely associated with higher incidence of hypertension, coronary heart disease, and diabetes, while reduced BMI, on the other hand, is also a sign of malnutrition and frailty status in the elderly (Graboyes et al., 2018; Sperling et al., 2018; Workman et al., 2020; Cutler et al., 2021). Hyperbilirubinemia reflects underlying hemolysis and hepatic dysfunction. Such patients have decreased tolerance for massive intraoperative blood loss, hypotension, and hepatic ischemia (Liao et al., 2013; Arvind et al., 2021). Elevated blood glucose level, usually including type 2 diabetes mellitus and impaired fasting glucose, is associated with postoperative infections, which are common causes of postoperative unplanned readmissions (Jones et al., 2017; Martin et al., 2019).

To improve the performance of unplanned readmission risk prediction, we combined the RF and XGBoost classifiers by setting weights according to MAE. Our study demonstrates that the combined model could perform significantly better than individual models in predicting unplanned readmission. Meanwhile, among all the models, MLP did not achieve relatively good scores, which may be because the neural network algorithm is relatively complex for small unbalanced text datasets. Actually, the performance of ML algorithms is closely related to the imbalance rate of a label (e.g., imbalance rate of unplanned readmission). When the number of positive samples is excessively low (<10%), ML algorithms are easily overfitted. In this study, the 30-day unplanned readmission rate was lower than 5%, indicating a high probability of predicting patients as negative samples. Although we used SMOTEENN as a sampling method to reduce the imbalance rate, the classification performance has much room for improvement, as seen from the recall and F1 scores. The Brier score of the hybrid model is 0.0372 (95% CI, 0.0371–0.0372), which is also the lowest among all the algorithms.

Our analysis of postoperative patients provides us with three key insights into the prediction of unplanned readmission. First, ML is a powerful artificial intelligence approach to using data to imitate the way that humans learn and make decisions, gradually improving its accuracy. In this study, nearly all models achieved an AUC of more than 0.7, whereas studies predicting unplanned readmissions achieved AUC in the range of 0.54–0.92 (Artetxe et al., 2018). Second, hybrid models may perform better than individual models. Third, effective data processing is essential to assist decision-making. Strategies to reduce potentially avoidable 30-day readmissions may help improve the quality of care and outcomes.

### Limitations

Some potential limitations should be considered. First, we did not include the information of hospital personnel for analysis. There is no doubt it is closely related to the patients’ outcome; second, this is a monocenter study, and most of the patients came from western China. As a result, further external validation is needed. Third, the sample size is relatively small compared to some retrospective studies. Fourth, during data collection and follow-up, it is inevitable that some data will be missing.

## Conclusion

ML algorithms can accurately predict postoperative unplanned 30-day readmission in elderly surgical patients.

## Data Availability

The raw data supporting the conclusion of this article will be made available by the authors, without undue reservation.
